# The use of high-efficacy disease-modifying therapies in multiple sclerosis: recommendations from an expert Delphi consensus

**DOI:** 10.1007/s00415-025-13293-9

**Published:** 2025-08-10

**Authors:** Massimo Filippi, Maria Pia Amato, Diego Centonze, Paolo Gallo, Claudio Gasperini, Matilde Inglese, Francesco Patti, Carlo Pozzilli, Paolo Preziosa, Maria Trojano

**Affiliations:** 1 Neuroimaging Research Unit, Division of Neuroscience, IRCCS San Raffaele Scientific Institute, Milan, Italy; 2 Neurology Unit, IRCCS San Raffaele Scientific Institute, Via Olgettina, 60, 20132 Milan, Italy; 3 Neurorehabilitation Unit, IRCCS San Raffaele Scientific Institute, Milan, Italy; 4 Neurophysiology Service, IRCCS San Raffaele Scientific Institute, Milan, Italy; 5 Vita-Salute San Raffaele University, Milan, Italy; 6 Department NEUROFARBA, University of Florence, Florence, Italy; 7 IRCCS Fondazione Don Carlo Gnocchi, Florence, Italy; 8 Department of Systems Medicine, Tor Vergata University, Rome, Italy; 9 IRCCS Neuromed, Pozzilli, Isernia Italy; 10 Department of Neuroscience, University of Padova, Padova, Italy; 11 Azienda Ospedaliera of Padua, Padua, Italy; 12 Department of Neurosciences, S Camillo Forlanini Hospital Rome, Rome, Italy; 13 Department of Neuroscience, Rehabilitation, Ophthalmology, Genetics, Maternal and Child Health (DINOGMI), University of Genoa, Genoa, Italy; 14 IRCCS Ospedale Policlinico San Martino, Genoa, Italy; 15 Department of Medical and Surgical Sciences and Advanced Technologies “G.F. Ingrassia”, University of Catania, Catania, Italy; 16 Azienda Ospedaliero-Universitaria Policlinico “G. Rodolico-S. Marco”, Catania, Italy; 17 S. Andrea MS Center, Sapienza University, Rome, Italy; 18 University of Bari “Aldo Moro”, Bari, Italy

**Keywords:** Multiple sclerosis, Treatment, Delphi

## Abstract

**Objective:**

To establish recommendations based on an expert consensus on the early and appropriate use of high-efficacy disease-modifying therapies (HE-DMTs) in the management of multiple sclerosis (MS) patients, based on current clinical evidence and real-world practice in Italy.

**Material and methods:**

A Delphi panel comprising 65 neurologists from 54 Italian MS centers engaged in a two-round consensus process. Experts rated 43 statements across five domains: therapeutic goals, definitions of HE-DMT, MS patient profiling, and use of HE-DMT at diagnosis and later in MS course, using a 5-point Likert scale. A statement reached strong consensus if ≥80% of panelists agreed; whereas between 70% and 80% it was considered as moderate.

**Results:**

In Round 2, 53 experts completed the survey on 43 statements. Strong consensus was achieved for 33 (76.7%), and moderate consensus for 6 (14.0%) statements. Experts strongly supported early HE-DMT initiation to prevent irreversible disability, endorsed a multidimensional definitions of treatment efficacy, and recommended personalized approaches based on clinical, radiological, and biomarker indicators. Consensus supported initiating HE-DMTs in patients with poor prognostic features and identified magnetic resonance imaging (MRI) activity, neurodegeneration markers, and suboptimal clinical response as specific factors requiring escalation to HE-DMTs.

**Conclusion:**

This Italian Delphi underscores the importance of early, personalized HE-DMT use to optimize long-term outcomes in MS. The strong expert alignment reflects a paradigm shift toward proactive treatment and highlights actionable clinical, radiological, and biological indicators that should guide therapeutic decisions. These findings may support national policy changes and promote more equitable and evidence-based access to HE-DMTs across healthcare systems.

## Introduction

Multiple sclerosis (MS) is a chronic, progressive, immune-mediated disorder of the central nervous system (CNS), characterized by neuroinflammation, demyelination, and neurodegeneration [1]. Pathophysiological processes often begin early, even before clinical symptoms become evident [1], highlighting the need for timely therapeutic intervention to prevent progression to irreversible damage and disability [2–6].

In recent years, the treatment landscape for MS has evolved substantially with the advent of high-efficacy (HE) disease-modifying therapies (DMTs) [2, 4, 6–14]. DMTs are commonly classified as moderate-efficacy (ME)-DMTs or HE-DMTs [2, 4, 6–13, 15]. While therapeutic strategies have advanced, the optimal timing and patient selection for initiating HE-DMTs remains a subject of ongoing debate [2, 4, 7–13, 16]. HE-DMTs consistently outperform ME-DMTs in reducing relapse rates, delaying disability progression, and limiting CNS damage accumulation in terms of focal lesions and irreversible tissue loss [6, 17–23]. However, HE-DMTs are typically administered only in MS patients with highly active disease whereas their use is often been postponed until after ME-DMT failure in many MS patients, resulting in delays in achieving optimal disease control and potentially compromising long-term outcomes [2, 4, 6–14, 24, 25]. Growing evidence now supports the early use of HE-DMTs to mitigate inflammatory and neurodegenerative processes, preserve neurological function, and improve long-term prognosis, challenging the escalation approach [2, 5, 6, 17–23, 26]. Despite emerging evidence, key questions remain regarding patient selection, risk stratification, and the long-term safety, underscoring the need for expert consensus and guidance to support clinical practice [2, 3, 6, 27, 28].

To address these gaps and support evidence-based clinical decision-making, a two-round Delphi consensus process was conducted involving 65 MS specialists from 54 Italian MS centers. This manuscript presents the key findings and expert consensus aimed at optimizing HE-DMT use in clinical practice and harmonizing treatment strategies across Italy.

## Materials and methods

### Study design and Delphi methodology

This study employed the Delphi method to gather and synthesize expert opinions on the appropriate use of HE-DMTs in the treatment of MS patients in Italy. The goal was to address current areas of clinical uncertainty, barriers and practice variability and barriers that may delay the timely adoption of HE-DMTs in clinical practice. The Delphi process was conducted according to established literature-based methodological procedures [29].

**Fig. 1:**
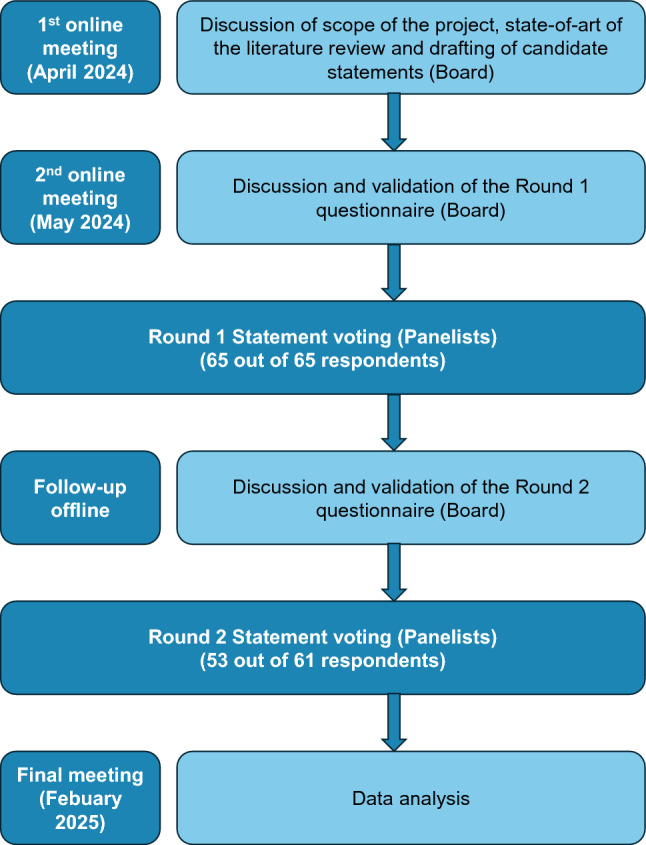
Delphi process flow diagram. Schematic representation of the different phases of the Delphi-method-based process

A Scientific Board comprising nine neurologists with great expertise in MS patients’ treatment was appointed at the outset of the project. Sixty-five MS specialists from 54 major Italian MS centers were invited to join in the Delphi panel and participate in the consensus process (Fig. 1).

MS centers were selected according to the number of MS patients under management, with at least 500 MS patients being treated, as recorded in the Italian MS and Related Disorders (I-MS&RD) Register (accessed April 2024) [30]. The participating centers were categorized as follows: 12 centers managing 500–1,000 MS patients, 17 managing 1000–2000 MS patients, and 6 managing over 2000 MS patients. Altogether, these centers care for a total of 54,546 MS patients. Centers were also selected to ensure broad geographic representation across the national territory.

During the first meeting, the Scientific Board defined the project scope and drafted a preliminary set of candidate statements for the Round 1 questionnaire based on current state-of-the art and clinical practice. The Round 1 questionnaire was then revised for the first round of analysis to minimize redundancies, as well as to ensure clear and consistent wording. The final Round 1 questionnaire included 43 statements distributed across five main categories (Table 1): (1) Therapeutic goals in MS and best treatment strategies; (2) HE-DMTs: definition of “high efficacy” and supporting evidence for their early use; (3) Treatment strategies based on patients’ profiles; (4) Factors influencing HE-DMT initiation at diagnosis; (5) Factors influencing late HE-DMT use.

**Table 1:** The statements of the Delphi questionnaire “HE-DMT makes the difference”

N.	Statement	Consensus level		
		Lack of consensus (<70%)	Moderate consensus (70≤ X <80%)	Strong consensus (≥80%)
*(1) Therapeutic goals in MS and best treatment strategy to reach them*				
S1	MS is characterized by neuroinflammation and neurodegeneration that may be underestimated			**98.5%**
S2	Treatment goals consist in hindering the underlying pathophysiological mechanisms (i.e., inflammation and neurodegeneration) early in the disease course preventing the progression of irreversible disability			**100.0%**
S3	Early initiation of an HE-DMT could be associated with a better risk/benefit ratio vs an escalation approach, which is often associated with a lack of disease control			**92.3%**
*(2) HE-DMTs: defining high efficacy and supporting evidence for their early use*				
S4	A therapy can be defined as HE-DMT if a higher reduction vs relative comparator in pivotal studies (be it an active comparator or placebo) can be proven on: • >1 outcome of inflammation: - Substantial decrease (>45–50%) of ARR - Relative reduction (≥80%) of MRI activity (new/enlarging T2-hyperintense WM lesions and/or Gd-enhancing lesions AND • ≥1 outcome of disease progression: - Substantial higher decrease of clinical disability progression: confirmed worsening of EDSS score and its functional system scores, and/or cognitive deterioration, and/or composite scores (e.g., MSFC, EDSS worsening plus ≥ 20% minimum threshold change for T25FWT and 9HPT) - Substantial effect on MRI measures of neurodegeneration: global or regional brain and spinal cord atrophy - Substantial effect on body fluid biomarkers: NfL levels - PROs			**89.2%**
*(3) Treatment strategies based on patients’ profiles*				
S5	The treatment with a HE-DMT is advisable to the vast majority of patients and it is not contraindicated for the other ones			**83.0%**
S6	It is mandatory to offer early treatment initiation with an HE-DMT when prognostic factors indicate aggressive disease (see statements from 8 to 23)			**100.0%**
S7	When evaluating treatment options, patient-related factors such as comorbidities, preferences, and family planning should be considered			**100.0%**
*(4) Factors bringing to the use of HE-DMT at the diagnosis* *A HE-DMT is advisable for all patients. In particular, its prescription is mandatory for patients who exhibit the following demographic factors:*				
S8	Although demographic and environmental factors (e.g., non-Caucasian, male sex, smoking) could lead to a worse prognosis, they should not be considered as relevant to decide whether to start a HE-DMT.	**39.6%**		
S9	Demographic and environmental factors, such as older age AND/OR obesity should be taken into account when a HE-DMT therapy is considered.			**87.7%**
*A HE-DMT is advisable for all patients. In particular, its prescription is mandatory for patients who exhibit the following clinical factors:*				
S10	Onset with documented motor and/or cerebellar and/or bladder/bowel symptoms			**89.2%**
S11	Short inter-attack latency (less than 3 months)			**96.9%**
S12	Multifocal onset (≥2 functional systems involved simultaneously)			**98.5%**
S13	Documented cognitive impairment			**81.5%**
S14	Incomplete recovery after a relapse documented by EDSS assessment			**93.8%**
*A HE-DMT is advisable for all patients. In particular, its prescription is strongly recommended for patients who exhibit the following biochemical factors:*				
S15	Presence of cerebrospinal OCBs	**49.1%**		
S16	Elevated NfL levels in adults suggest aggressive disease activity, as confirmed by MRI scans		**75.5%**	
*A HE-DMT is advisable for all patients. In particular, its prescription is mandatory for patients who exhibit the following neuroradiological factors:*				
S17	Brainstem and cerebellar lesions			**93.8%**
S18	Spinal cord lesions (especially affecting the central GM)			**95.4%**
S19	Cortical lesions			**83.1%**
S20	At least 2 Gd-enhancing lesions		**75.5%**	
S21	Chronic active lesions (PRL or SELs)			**84.9%**
S22	Brain atrophy (especially GM)		**79.2%**	
S23	Spinal cord atrophy (especially GM)			**83.1%**
*(5) Factors bringing to the use of HE-DMT at the follow up later on in the disease* A switch to an HE-DMT is advisable for all patients. In particular, it is mandatory for patients who exhibit the following clinical factors:				
S24	New onset or worsening of motor and/or cerebellar and/or bladder/bowel symptoms			**96.9%**
S25	A new relapse occurring within the first 6 months after treatment initiation		**77.4%**	
S26	Short inter-attack latency (less than 3 months)			**93.8%**
S27	Incomplete recovery after a relapse documented by EDSS assessment			**89.1%**
S28	Severe clinical relapses			**98.4%**
S29	High disability accumulation in the first 2–5 years from disease onset			**93.8%**
S30	A new PIRA event more than 6 months from treatment initiation		**75.5%**	
S31	Worsening of cognitive impairment documented by neuropsychological testing, compared to the baseline		**73.6%**	
S32	Continued disease activity despite DMT			**100.0%**
*A switch to a HE-DMT therapy is advisable for all patients. In particular, it is mandatory for patients who exhibit the following biochemical factors:*				
S33	Presence of CSF-specific OCBs	**34.0%**		
S34	High NfL levels (in adulthood), or an increase of NfL level compared to the baseline, suggesting suboptimal control of disease activity (confirmed by an MRI scan)			**83.0%**
*A switch to a HE-DMT therapy is advisable for all patients. In particular, it is mandatory for patients who exhibit the following neuroradiological factors:*				
S35	New T2-hyperintense WM lesions formation			**87.3%**
S36	Brainstem and cerebellar lesions			**81.0%**
S37	Spinal cord lesions (especially affecting the central GM)			**87.3%**
S38	Cortical lesions			**81.0%**
S39	Brain atrophy (especially GM)	**69.8%**		
S40	Spinal cord atrophy (especially GM)			**81.0%**
S41	Presence of Gd-enhancing lesions			**92.1%**
S42	Chronic active lesions (paramagnetic iron rim or slowly expanding)			**84.1%**
*Factors bringing to the use of HE-DMT at the follow up later on in the disease*				
S43	Please indicate your agreement that the following are the most important factors that should prompt an early switch to HE-DMT: - new onset or worsening of motor and/or cerebellar and/or bladder/bowel symptoms - incomplete recovery after a new relapse documented by EDSS assessment - worsening of cognitive impairment documented by neuropsychological testing, compared to the baseline - a new T2-hyperintense WM lesion (especially in spinal cord, brainstem, or cerebellum) - a new Gd-enhancing lesion			**98.1%**

*9HPT* Nine-Hole Peg Test; *ARR* annualized relapse rate; *CSF* cerebrospinal fluid; *EDSS* Expanded Disability Status Scale; *Gd* gadolinium; *GM* gray matter; *HE-DMT* high-efficacy disease-modifying therapy; *MRI* magnetic resonance imaging; *MS* multiple sclerosis; *MSFC* Multiple Sclerosis Functional Composite; *NfL* neurofilament light chain; *PIRA* progression independent of relapse activity; *PRL* paramagnetic rim lesion; *PROs* patient’s reported outcomes; *SEL* slowly-expanding lesion; *T25FWT* Timed 25-Foot Walk test; *WM* white matter

Each statement was rated using a 5-point Likert scale [26, 31–34]: 1 (“strongly disagree”), 2 (“disagree”), 3 (“slightly agree”), 4 (“agree”), and 5 (“strongly agree”). During Round 1, the panelists could also provide open-ended comments on each statement. A total of sixty-five members of the Panel belonging to 54 centres completed the round 1 questionnaire.

Consensus thresholds were predefined a priori. A statement was considered to have reached a “strong” consensus if ≥80% of panelists selected ratings of 4 or 5. The Scientific Board further defined that an agreement between 70% and 80% should be considered as “moderate” consensus, whereas statements with <70% agreement were considered to have failed to reach consensus.

Following Round 1, results and qualitative feedback were reviewed by the Scientific Board, leading to minor revisions for Round 2. The panelists were then asked to vote the statements in the Round 2 questionnaire. Sixty-one (out of 65) panelists from 40 MS centers were invited to participate in the Round 2. A methodology expert supported data analysis and the development of the final report.

## Results

Fifty-three panelists out of 61 from 35 centers completed the second-round questionnaire, yielding a participation rate of 86.8%.

Of the 43 statements evaluated in Round 2, 33 (76.7%) achieved strong consensus (≥80%) and 6 (14.0%) reached moderate consensus (70–79%) (Table 1).

### Therapeutic goals in MS and best treatment strategies to reach them

All statements within this category reached strong consensus (Table 1). Experts largely agreed (98.5%) that MS involves both neuroinflammatory and neurodegenerative mechanisms, which are often underestimated (Statement S1). There was unanimous agreement (100%) that therapeutic strategies should target these mechanisms early in the disease course to prevent the progression of irreversible disability (100%) (Statement S2). Furthermore, 92.3% of panelists endorsed the notion that early initiation of HE-DMTs offers a more favorable risk-benefit profile compared to the traditional escalation approach, which is frequently associated with suboptimal disease control (Statement S3).

### HE-DMTs: definition of “high efficacy” and supporting evidence for their early use

**Fig. 2:**
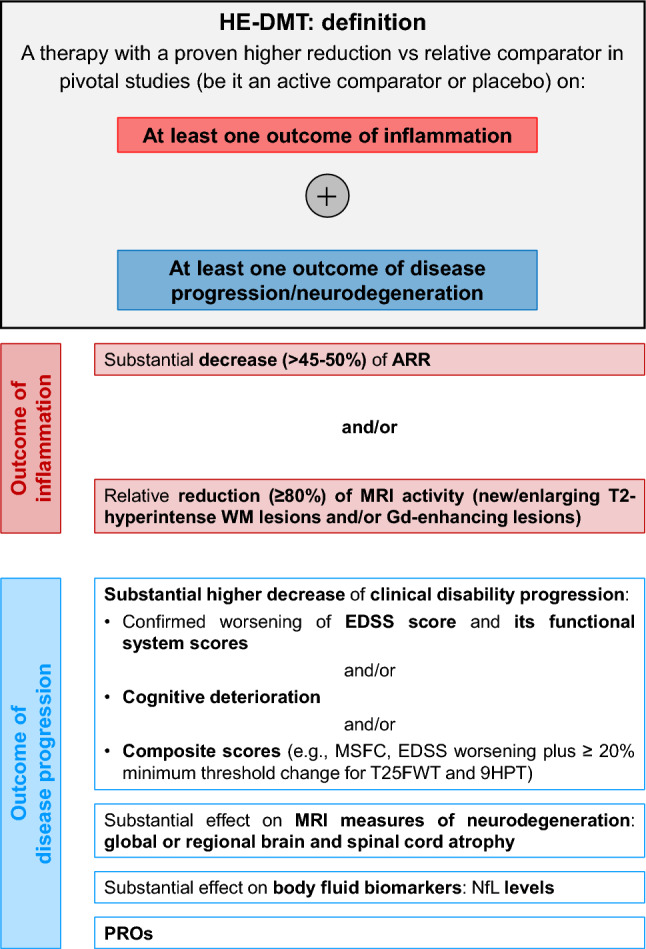
Features defining a DMT as HE for MS patients. *9HPT* = Nine-Hole Peg Test; *ARR* annualized relapse rate; *EDSS* Expanded Disability Status Scale; *Gd* gadolinium; *HE-DMT* high-efficacy disease-modifying therapy; *MRI* magnetic resonance imaging; *MS* multiple sclerosis; *MSFC* Multiple Sclerosis Functional Composite; *NfL* neurofilament light chain; *PROs* patient’s reported outcomes; *T25FWT* Timed 25-Foot Walk test; *WM* white matter

A strong consensus (89.2%) was reached on the criteria that define a DMT as HE (Statement S4) (Table 1, Fig. 2).

A therapy can be defined as HE-DMT if a higher reduction vs relative comparator in pivotal studies (be it an active comparator or placebo) can be proven on:

- >1 outcome of inflammation:
  - Substantial decrease (≥45–50% reduction) of the annualized relapse rate (ARR);
  - Relative reduction (≥ 80%) of magnetic resonance imaging (MRI) activity (new/enlarging T2-hyperintense WM lesions and/or Gd-enhancing lesions.

- ≥1 outcome of disease progression:
  - Substantial higher decrease of clinical disability progression: confirmed worsening of Expanded Disability Status Scale (EDSS) score and its functional system scores. and/or cognitive deterioration, and/or composite scores (e.g., Multiple Sclerosis Functional Composite [MSFC], EDSS worsening plus ≥ 20% minimum threshold change for Timed 25-Foot Walk test [T25FWT] and Nine-Hole Peg Test [9HPT]);
  - Substantial effect on MRI measures of neurodegeneration: global or regional brain and spinal cord atrophy;
  - Substantial effect on body fluid biomarkers: neurofilament light chain (NfL) levels;
  - Patient’s reported outcomes (PROs).

Experts agreed that a DMT may be considered HE if it demonstrates a significant impact on multiple dimensions, including inflammatory activity, disease progression, neurodegenerative MRI markers, fluid biomarkers, and PROs.

### Treatment strategies based on patients’ profiles

All statements in this category reached strong consensus (Table 1). The majority of panelists (83.0%) agreed that HE-DMTs are suitable for most patients and should not be considered contraindicated in others without specific clinical reasons (Statement S5), acknowledging the potential for more widespread applicability of HE-DMTs in diverse patient profiles.

Unanimous consensus (100%) supported the use of HE-DMTs in patients with poor prognostic indicators and aggressive disease phenotypes (statement S6). Similarly, 100% of panelists agreed that treatment decisions should include individual factors such as comorbidities, family planning, and patient preferences (Statement S7).

### Factors influencing HE-DMT initiation at diagnosis

For this category, responses varied depending on the type of predictor, with the majority of statements reaching a strong (≥80%) or moderate agreement (between 70–80%) among panelists (Table 1). Some demographic and lifestyle factors, such as male sex or smoking, were not considered sufficient to guide the initiation of HE-DMTs (Statement S8; 39.6% agreement). In contrast, moderate consensus (87.7%) was achieved for including older age and obesity as factors influencing early use of HE-DMTs.

Strong consensus was reached on several clinical features at onset as indicators of aggressive disease and prompting HE-DMT initiation. These included motor, cerebellar, or sphincter involvement (Statement S10; 89.2%), short inter-attack intervals (Statement S11, 96.9%), multifocal onset (Statement S12, 98.5%), cognitive impairment (statement S13, 81.5%), and incomplete recovery from relapse (statement S14, 93.8%).

Panelists did not reach consensus on cerebrospinal fluid (CSF)-specific oligoclonal bands (OCBs) as a factor warranting HE-DMT initiation (Statement S15; 49.1%). Conversely, elevated serum NfL levels achieved moderate consensus as an indicator of more severe disease (Statement S16, 75.5%).

MRI features received strong endorsement as critical indicators for early HE-DMT use. Brainstem and cerebellar lesions (Statement S17, 93.8%), spinal cord lesions (Statement S18, 95.4%), cortical lesions (Statement S19, 83.1%), chronic active lesions (paramagnetic rim lesions [PRLs] or slowly-expanding lesions [SELs]) (Statement S21, consensus = 84.9%), and spinal cord atrophy (Statement S23, 83.1%) were all recognized as relevant predictors of a more aggressive disease course. The presence of at least two gadolinium (Gd)-enhancing lesions (Statement S20, 75.5%) and brain atrophy (Statement S22, 79.2%) were also considered relevant, although both reached only moderate consensus.

### Factors influencing late HE-DMT use

The panel reached strong consensus on a comprehensive set of clinical, biochemical, and radiological factors that should prompt escalation to HE-DMTs during follow-up (Table 1). Panelists strongly agreed that the onset or worsening of motor, cerebellar, or sphincter symptoms (Statement S24, 96.9%), short inter-attack intervals (<3 months) (Statement S26, 93.8%), incomplete recovery following relapse (Statement S27, 89.1%), severe relapses (Statement S28, 98.4%), high disability accumulation within 2–5 years from disease onset (Statement S29, 93.8%), and continued disease activity despite current DMT (Statement S32, 100.0%). Conversely, moderate consensus was reached on new relapses within the first six months of DMT start (Statement S25, 77.4%), a progression independent of relapse activity (PIRA) event occurring after six months from DMT initiation (Statement S30, 75.5%), and worsening cognitive function (Statement S31, 73.6%).

A strong consensus (83.0%) supported using high or increasing serum NfL levels, when confirmed by MRI, as a signal of suboptimal disease control (Statement S34, 83.0%). No consensus was achieved for the presence of CSF-specific OCBs (Statement S34, 34.0%).

MRI findings were also emphasized as critical markers for treatment escalation. Strong consensus was reached for new T2-hyperintense white matter (WM) lesions formation (Statement S35; 87.3%), the presence of lesions in the brainstem or cerebellum (Statement S36; 81.0%), spinal cord lesions, particularly affecting central gray matter (GM) (Statement S37; 87.3%), cortical lesions (Statement S38; 81.0%), Gd-enhancing lesions (Statement S41; 92.1%), chronic active lesions (PRLs or SELs) (Statement S42; 84.1%), and spinal cord atrophy (Statement S40; 81.0%). Although clinically relevant, brain atrophy (especially of GM) did not reach consensus (Statement S39; 69.8%).

Finally, a near-unanimous consensus (98.1%) was reached on a comprehensive summary statement (Statement S43) that identified the most actionable indicators to justify timely switching to a HE-DMT. These included worsening motor, cerebellar, or sphincter symptoms, incomplete recovery after a relapse, cognitive decline, and the appearance of new T2-hyperintense WM lesions (especially spinal cord, brainstem, or cerebellum) or Gd-enhancing lesions.

## Discussion

This Delphi consensus, involving 53 MS specialists from 35 Italian centers collectively managing over 54,000 MS patients, showed a strong national alignment regarding the early and strategic use of HE-DMTs in MS care. The achievement of strong consensus on 76.7% of the statements, and moderate consensus on an additional 14%, reveals a scientific and clinical maturity among Italian neurologists in the understanding of MS pathophysiology and a shared commitment to shifting MS management toward a more proactive, efficacy-focused and evidence-based [2, 5, 6, 17–23] approach in MS care to optimize early treatment strategies.

The decision to adopt a stringent consensus threshold (≥80%), exceeding typical Delphi standards [26, 31–34], reinforces the robustness and rigor of this process and reflects a deliberate effort by the Scientific Board that the recommendations have both scientific validity and clinical relevance.

### Therapeutic goals in MS and best treatment strategies to reach them

Unanimous agreement was reached on the dual pathophysiology of MS, i.e., neuroinflammation and neurodegeneration, which often begins before clinical symptoms manifest [1, 2, 35]. Panelists agreed that therapeutic goals should target these mechanisms to prevent irreversible neurological damage, underscoring the critical therapeutic window in early disease phases [1, 2, 6, 35]. Growing evidence shows that HE-DMTs offer superior benefits in limiting new lesion formation, relapse rates, and reducing disability progression and brain atrophy compared to ME-DMTs [2, 3, 6, 17–23, 36]. Moreover, long-term data from randomized clinical trials (RCTs) and real-world data show that early use of HE-DMTs yields better clinical outcomes than ME-DMTs and escalation strategies [17–23, 36], while maintaining a good safety profile [24, 28, 37–42].

In line with this, a near-unanimous agreement (92.3%) affirmed that HE-DMTs offer a more favourable risk-benefit profile when initiated early in the disease course.

### HE-DMTs: definition of “high efficacy” and supporting evidence for their early use

A strong consensus (89.2%) supported a broader, multidimensional definition of HE-DMTs [2, 3, 6], incorporating not only a substantial suppression of relapses (≥45–50% ARR reduction) and MRI activity (≥80% reduction in new/enlarging T2-hyperintense WM or Gd-enhancing lesions), but also effects on disability progression, brain/spinal cord atrophy, fluid biomarkers such as NfL, and PROs.

This expanded framework reflects evolving insights into disease progression and current challenges in MS management since overt inflammatory activity is often well-controlled by HE-DMTs, yet subclinical progression (i.e., progression independent of relapse activity [PIRA]), may still occur [16, 43–49].

Panelists also emphasized the importance of cognitive function [50] and PROs in efficacy assessment [25], which are increasingly recognized as critical outcomes in MS management, but may not be adequately captured by traditional clinical endpoints.

In addition, this widely accepted definition clarifies which DMTs are to be classified as HE-DMTs (natalizumab, alemtuzumab, ocrelizumab, ofatumumab and ublituximab).

Nevertheless, some concerns were raised regarding the rigidity of current classification systems that may undervalue therapies lacking head-to-head trial data, despite strong real-world efficacy. The panel emphasized the need for flexibility in interpreting efficacy, taking into account clinical judgment, patient variability, and access to biomarkers or advanced MRI analyses, which remain limited in many settings.

### Treatment strategies based on patients’ profiles

There was strong consensus (83.0%) that HE-DMTs are appropriate for the majority of MS patients and should not be discouraged in others without clear contraindications. Unanimous agreement supported the use of HE-DMTs for MS patients with negative prognostic indicators, such as early disability, multifocal onset, or aggressive relapse activity [15, 51]. Moreover, panelists highlighted the importance of considering comorbidities, reproductive planning, and patient preferences in therapeutic decisions. These findings align with personalized medicine principles and support models of shared decision-making known to improve treatment adherence, satisfaction, and long-term outcomes [52, 53].

### Factors influencing HE-DMT initiation at diagnosis

The Delphi panel made key distinctions between factors that are predictive of poor prognosis and those lacking sufficient evidence. Demographic and lifestyle variables such as sex and smoking did not reach consensus as features supporting early HE-DMT use, possibly due to the lack of clear evidence of a different effect of DMTs according to these factors. Conversely, older age and obesity were acknowledged as relevant (87.7%). These factors are recognized as negative prognostic factors [15], and they may also influence treatment response and MS trajectory [15, 54, 55]. For instance, HE-DMTs seem to be less effective in older individuals, likely due to reduced inflammatory activity and a shift toward neurodegenerative mechanisms.

Strong consensus supported the role of early clinical features that are well known predictors of aggressive disease, such as motor, cerebellar or sphincter involvement, short inter-attack latency, multifocal onset, cognitive impairment, and incomplete relapse recovery, as justifications for HE-DMT initiation [1, 15].

Similarly, several MRI features were endorsed as critical predictors of aggressive disease, including brainstem/cerebellar lesions, spinal cord lesions, cortical lesions, chronic active lesions (PRLs or SELs), and spinal cord atrophy. This is in line with the evidence that each of these findings is associated with a higher risk of clinical progression and cognitive decline [48, 51, 56]. Only moderate consensus was reached on brain atrophy, likely due to some practical barriers, including the lack of standardized cut-off values for defining pathologic atrophy in MS and limited access to volumetric MRI tools in routine practice. These limitations likely reduced the perceived reliability of brain atrophy as a factor for early HE-DMT initiation, despite robust evidence of its prognostic value [48, 51, 56, 57]. In contrast, spinal cord atrophy was more strongly endorsed, possibly because it is perceived as a more specific marker of aggressive disease, with clearer associations with clinical disability and fewer confounding factors such as aging.

CSF-specific OCBs did not reach consensus, whereas NfL reached only moderate consensus (75.5%). Even though the presence of CSF-specific OCBs represents a negative prognostic factor [1, 15], their association with DMT efficacy and selections has not been explored. While the prognostic potential of NfL is recognized [50, 58–60], NfL levels alone cannot guide therapeutic decisions and should be interpreted in conjunction with MRI findings. Furthermore, limited accessibility, inter-center variability in interpretation, and the absence of standardized normative values further constrain their utility in guiding DMT selection.

### Factors influencing late HE-DMT use

Strong consensus was reached for clinical signs warranting escalation to HE-DMTs, including new or worsening of motor, cerebellar, or sphincter symptoms, severe relapses, short inter-attack intervals, incomplete recovery from relapse, and early disability accumulation. Continued disease activity despite current DMTs garnered unanimous agreement, reinforcing the need for timely therapeutic reassessment.

Only moderate consensus was achieved for new relapses within 6 months of DMT initiation, PIRA events occurring after 6 months from DMT start, and cognitive decline. Relapses within the first six months may reflect ongoing disease activity not yet controlled by DMTs, as they often require several weeks to months to achieve their full therapeutic effect. Therefore, such relapses are not necessarily indicative of treatment failure. Both PIRA and cognitive impairment are increasingly recognized as markers of subclinical disease progression [47, 48, 56]. However, the moderate consensus for PIRA and cognitive worsening may reflect practical challenges in reliably assessing these outcomes in clinical settings, the need for a longitudinal monitoring to detect them, and the apparent limited impact of HE-DMTs on these outcomes [43, 50].

NfL was recognized as a promising biomarker for monitoring disease activity and therapeutic response [50, 58–60], with strong consensus supporting its use when interpreted in conjunction with MRI findings.

MRI remains a cornerstone of MS follow-up, with strong consensus supporting the importance of new T2 lesions, Gd-enhancing lesions, lesions in eloquent regions (e.g., brainstem, cerebellum, spinal cord, and cortex), chronic active lesions, and spinal cord atrophy as indicators for treatment escalation.

These findings reinforce the utility of MRI not only in detecting inflammatory activity, but also in capturing neurodegenerative changes associated with silent disease progression or PIRA [44, 46–48, 51, 56, 57].

A near-unanimous consensus supported a composite summary statement integrating key clinical (i.e., new onset/worsening of motor, cerebellar, or sphincter symptoms, incomplete recovery from relapse, cognitive deterioration) and MRI features (new T2-hyperintense WM or Gd-enhancing lesions) to guide timely switching to HE-DMTs. These features were deliberately selected for their feasibility, relevance, and applicability across different clinical settings, including those without access to advanced imaging or biomarker testing.

## Limitations

Despite its strengths, the Delphi method is inherently limited by expert selection and potential biases in subjective interpretation. Furthermore, while the consensus reflects a representative cross-section of Italian MS centers, variations in resource access (e.g., biomarkers, advanced MRI data) may limit generalizability across healthcare systems. Additionally, although the panel included a broad and representative sample of high-volume MS centers across Italy, we acknowledge that the views of neurologists not included in this Delphi process, particularly those practicing in smaller centers or with differing organizational models, may diverge from those presented here. Future studies involving a more heterogeneous sample of clinicians could help assess the external validity and broader applicability of these consensus recommendations.

Furthermore, while the structured use of Likert scales facilitates standardization and quantification of consensus, it may also obscure more nuanced differences in expert opinion that could emerge in less constrained formats such as qualitative interviews or focus groups. Given that several recommendations—especially those concerning HE-DMT safety profiles, fluid biomarkers such as serum NfL, and advanced MRI features—are based on emerging or rapidly evolving evidence, periodic re-evaluation of these consensus statements will be essential to ensure continued alignment with the latest clinical research.

Finally, in future Delphi initiatives, further efforts should be made to pre-validate the semantic clarity of each statement, as subtle ambiguities in phrasing (e.g., ‘not contraindicated’ vs. ‘not discouraged’) may influence the level of agreement. Combining quantitative Delphi rounds with qualitative methods (e.g., focus groups) could further enhance interpretability and consensus fidelity. Additionally, the involvement of expert linguists in reviewing the proposed statements may help identify potential inaccuracies or imprecisions in terminology, thereby improving consistency and reducing the risk of misinterpretation.

## Conclusions

This national Delphi consensus establishes comprehensive, expert-driven recommendations supporting early and individualized use of HE-DMTs in MS clinical practice, underpinned by clinical and radiological indicators and grounded in both RCT data and real-world experience. The integration of emerging biomarkers, such as NfL, chronic active lesions and brain/spinal cord atrophy, into clinical frameworks may further optimize long-term outcomes.

The resulting consensus statements reflect not only theoretical considerations, but also practical insights drawn from routine clinical practice. Moreover, the process reveals the maturity of the Italian MS care network and its readiness to implement evidence-based strategies in everyday clinical settings. By identifying widely accepted clinical and radiological indicators, this consensus provides a foundation for more consistent, appropriate and equitable access to HE-DMTs nationwide.

Nonetheless, challenges remain in translating consensus into practice. In Italy, current prescribing restrictions and rigid reimbursement policies continue to hinder access to HE-DMTs. These constraints can limit clinical autonomy and may compromise optimal patient care. Adopting more flexible, risk-adapted prescribing models, allowing neurologists to initiate any approved DMT without the prerequisite failure of a moderate-efficacy therapy, would better align clinical practice with the latest scientific evidence and principles of patient-centered care.

However, to address the methodological and implementation challenges highlighted in this consensus, the Italian MS community is actively pursuing several coordinated strategies. These include the enhanced integration of the Italian MS and Related Disorders (I-MS&RD) Register to support real-world monitoring of therapeutic choices and outcomes, and National efforts to improve access to advanced diagnostic tools—such as volumetric MRI protocols and fluid biomarkers (e.g., serum NfL)—through multicenter collaboration and shared infrastructure. In parallel, the development and dissemination of standardized clinical care pathways (Percorsi Diagnostico Terapeutici Assistenziali, PDTA) [61] aim to ensure a more uniform and timely adoption of HE-DMTs across diverse healthcare settings. Furthermore, future Delphi processes will benefit from additional methodological refinements, including linguistic review of consensus statements by experts in medical communication to eliminate semantic ambiguities, and the integration of qualitative methodologies (e.g., interviews or focus groups) to better capture complex or context-dependent clinical judgments.

## Contributors

Umberto Aguglia––Department of Medical and Surgical Sciences, Magna Graecia University of Catanzaro, Catanzaro; Pietro Annovazzi––ASST Valle Olona, Gallarate (VA); Carlo Avolio––Università Foggia/Policlinico Foggia, Foggia; Viola Baione––Sapienza Università di Roma, Roma; Giacomo Boffa––Università di Genova, Genova; Paola Cavalla––Dipartimento di Neuroscienze “Rita Levi Montalcini”, Università di Torino, Torino; Raffaella Cerqua––Clinica Neurologica, Azienda Ospedaliero Universitaria delle Marche, Ancona; Clara Chisari––Università degli studi di Catania, Catania; Eleonora Cocco––University of Cagliari, Cagliari; Elena Colombo––IRCCS Fondazione Mondino, Pavia; Maria Gabriella Coniglio––P.O. Madonna delle Grazie, Matera; Antonella Conte––Sapienza University of Rome, Rome; Giovanns De Luca––Policlinico SS Annunziata Clinica Neurologica, Chieti; Alessia Di Sapio––Regional Referral Multiple Sclerosis Centre (CReSM), Department of Neurology, University Hospital San Luigi Gonzaga, Orbassano (TO); Roberta Fantozzi––IRCCS NEUROMED, Pozzilli (IS); Elisabetta Ferraro––Ospedale San Filippo Neri, Roma; Diana Ferraro––Ospedale Civile di Baggiovara, Azienda Ospedaliero-Universitaria di Modena, Modena; Matteo Foschi––Department of Neuroscience, Multiple Sclerosis Center, S. Maria delle Croci Hospital, AUSL Romagna, Ravenna; Jessica Frau––Centro Sclerosi Multipla Ospedale Binaghi-Università di Cagliari, Cagliari; Maurizia Gatto––Ospedale Generale Regionale Miulli, Acquaviva delle Fonti (Bari); Franco Granella––Università di Parma, Parma; Clara Guaschino––U/O Neurologia ad indirizzo neuroimmunologico, Centro Sclerosi Multipla, Gallarate (VA); Shalom Haggiag––Azienda Ospedaliera San Camillo Forlanini, Roma; Pietro Iaffaldano - Dipartimento di Biomedicina Traslazionale e Neuroscienze, Università degli Studi di Bari Aldo Moro, Bari; Antonio Ianniello––Department of Human Neurosciences, Sapienza University of Rome, Rome; Caterina Lapucci––IRCCS Ospedale Policlinico San Martino, Genova; Lorena Lorefice––Multiple Sclerosis Centre, PO Binaghi, University of Cagliari, ASL Cagliari, Cagliari; Matteo Lucchini––Fondazione Policlinico Universitario Agostino Gemelli IRCCS; Università Cattolica del Sacro Cuore, Roma; Alessia Manni––Università degli Studi di Bari “Aldo Moro”, Bari; Girolama Alessandra Marfia––Dipartimento Medicina dei Sistemi, Università Tor Vergata, Roma; Massimiliano Mirabella––UO Sclerosi Multipla, Fondazione Policlinico Universitario “A. Gemelli” IRCCS, Università Cattolica del Sacro Cuore, Roma; Lucia Moiola––IRCCS Ospedale San Raffaele, Milano; Damiano Paolicelli––Università degli Studi di Bari, Dipartimento di Biomedicina Traslazionale, DIBraiN, Bari; Maria Barbara Pasanisi––IRCCS Don C. Gnocchi Foundation ONLUS, Milano; Paola Perini––Clinica Neurologica, Azienda Ospedale Università Padova, Padova; Maria Grazia Piscaglia––Santa Maria Delle Croci, Hospital, Ravenna; Paolo Preziosa––IRCCS Ospedale San Raffaele, Università Vita-Salute San Raffaele, Milano; Alessandra Protti––ASST Grande Ospedale Metropolitano Niguarda, Milano; Paolo Ragonese––Dipartimento di Biomedicina, Neuroscienze e Diagnostica avanzata; Università di Palermo, Palermo; Eleonora Rigoni––IRCCS Fondazione Mondino, Pavia; Francesca Rinaldi––Azienda Ospedaliera, Università di Padova, Padova; Maria Assunta Rocca––IRCCS Ospedale San Raffaele, Università Vita-Salute San Raffaele, Milano; Marco Rovaris––IRCCS Fondazione Don Carlo Gnocchi, Milano; Andrea Surcinelli––Department of Neuroscience, MS Center, Neurology Unit, S. Maria delle Croci Hospital of Ravenna, Ravenna; Valentina Tomassini––Dept of Neurosciences, Imaging and Clinical Sciences, University G. d’Annunzio of Chieti-Pescara, Chieti; Valentina Torri Clerici––Istituto Neurologico Carlo Besta, Milano; Carla Tortorella––San Camillo Forlanini Hospital, Roma; Rocco Totaro––Centro Malattie Demielinizzanti-Ospedale San Salvatore, L’Aquila; Domizia Vecchio––Università Piemonte Orientale, Novara; Marika Vianello––UO Neurologia, Ca’ Foncello Hospital, Treviso; Luigi Zuliani––Ospedale San Bortolo, Azienda ULSS8 Berica, Vicenza

## Data Availability

The corresponding author, who had complete access to all the data of the study, assumes responsibility for the integrity of the data and accuracy in the analysis. The anonymized data set used and analyzed for this study can be obtained from the corresponding author on reasonable request.
